# Examining the relationships between income inequalities and different dimensions of well-being in selected Central Eastern European (CEE) countries

**DOI:** 10.1371/journal.pone.0250469

**Published:** 2021-04-28

**Authors:** Małgorzata Szczepaniak, Andrzej Geise

**Affiliations:** 1 Department of Economics, Faculty of Economic Sciences and Management, Nicolaus Copernicus University in Torun, Toruń, Poland; 2 Department of Econometrics and Statistics, Faculty of Economic Sciences and Management, Nicolaus Copernicus University in Torun, Toruń, Poland; Szechenyi Istvan University, HUNGARY

## Abstract

This article examines the relationships between different dimensions of well-being and income inequalities across selected Central Eastern European countries after joining the European Union in 2004. Regarding the multivariety of well-being concept, it explores its 5 dimensions (material dimension, health dimension, education dimension, environmental dimension, happiness). Accounting for the interactions between dimensions of well-being matters for the inequalities, we conducted an in-depth analysis by adopting PMG estimation and panel ARDL model to assess the short-run and long-run links between variables. The results of conducted analysis allowed us to identify the canals through which income inequalities are linked directly or indirectly with the particular dimensions of well-being. In the long run, all the dimensions of well-being significantly affected income inequalities, and income inequality shaped material dimension, health, education, natural environment, and happiness. However, in the short run, the only dimension that shaped income inequalities was education. Income inequalities directly affected both health dimension and happiness.

## Introduction

Well-being is a multidimensional phenomenon [1, 2] that requires consideration of many dimensions of well-being, which are beyond the standard of living concept [3]. The analysis of well-being should explore, among others, objective dimensions, such as material, health, education, and environmental [4]. What is more, to provide a more holistic measure of well-being, subjective well-being, as measured by overall life satisfaction or happiness, should be included when social progress is measured [5, 6].

Income inequalities may have important consequences in many spheres of life, beyond the obvious monetary dimension, above all with regard to social cohesion [7]. What is more, income inequality not only is instrumental to reach social objectives, but also is a matter of social concern itself [1], being an important feature of distributive justice [8]. Because of a multi-faceted construct of well-being, its various components show different relations with income inequalities. Therefore, studies focusing on a general measure, for example, overall well-being or only subjective well-being may not uncover the real relations between income inequalities and various well-being components [9]. The results of the analysis of links between income inequalities and well-being are important to improve “well-being for all” [10].

As mentioned, the motivation for this research was to do a comprehensive study, which will allow a better understanding of the relationships between income inequalities and different dimensions of well-being. Taking into account interactions between well-being dimensions is of particular importance because this may prioritize different policy suggestions to improve the multidimensional well-being in CEE (Central Eastern European) countries. This article aims to identify the links among unequal distribution of income and 5 dimensions of well-being (material dimension, health dimension, education dimension, natural environment, and happiness). It is particularly interesting in this context to carry out analyses for the group of Central and Eastern European countries for which inequality issues are particularly important in the process of economic transformation, and these relationships are not yet widely identified. We analyzed 8 Central Eastern European countries (former communist countries, transition countries): Poland, Czech Republic, Slovakia, and Hungary, Baltic Sea countries: Lithuania, Latvia, Estonia, and Slovenia. The research period was from 2004 (after joining the European Union) to 2018.

Overall, the contributions of this article are twofold. First, this article offers multidimensional well-being and income inequality measures for CEE countries, which goes beyond the GDP per capita comparisons, by integrating more dimensions into analysis at the same time. Second, we conduct the in-depth analysis on the impact of well-being on income inequalities, as well as an analysis between the dimensions of well-being. In particular, we use panel ARDL (q, p, p, …, p) model to assess the short-run and long-run links between income inequalities and different dimensions of well-being and identify the canals through which income inequalities are linked directly or indirectly with material, health, educational, environmental, and happiness dimensions of well-being. To achieve the goal of this study, we use panel data method that take cross-sectional dependence between countries into account, which allows to avoid the low power and size distortions for the test used in the research.

The remainder of this article is organized as follows. Section II discusses the literature concerning the relationships between income inequality and well-being. The data and methods of analysis are described in section III and section IV, respectively. The results of the empirical analysis are presented in section V. The last section offers the concluding remarks.

## Relationships between inequality and well-being in its different dimensions—Literature review

CEE countries have undergone profound political and socioeconomic transformation since the demise of communist rule. They are grouped in the literature as the postsocialist welfare state. However, in the Esping–Andersen’s original welfare state regime typology, the CEE countries were not included [11]. They share certain features of the social-democratic model, in particular, the concern for equality of living condition and the preference for government intervention to achieve these results [10]. At the beginning of the transition, these countries were characterized with growing inequalities, which was seen as particularly problematic likely because former socialist states had stronger preferences for equality or at least a long trajectory of fairly equitable distributions [12]. After joining the European Union in 2004, the CEE countries have been characterized by relatively low level of well-being measures both subjective well-being and non-income dimensions of well-being [13] in comparison to liberal and conservative-corporatist European countries. It is suggested that it is likely because of the low inequality in the market income distribution rather than the redistributive activities of their current welfare states [10]. What is more, the socioeconomic transformation processes in CEE countries brought many social problems before, that is why the advances in well-being made in post–socialist countries have been more limited than, suppose, on GDP growth [14, 15]. Although different welfare state regimes achieve different outcomes in dealing with social inequalities and differently affect well-being at the same time [16], there is a lack of such analysis for the CEE countries. To our knowledge, the only exemption is the study conducted by Brzeziński; however, it focused on factors that contributed to changes in income inequalities in CEE countries and covered the period of economic crisis between 2008 and 2012 [17].

There is a commonly held belief in the majority of literature that inequality has negative consequences for an individual’s health, education, and ultimately, lower well-being [18]. However, the empirical findings of researches regarding the connection between income inequalities and well-being are inconclusive. Furthermore, the analyses often focus on particular dimensions of well-being and income inequalities.

When relationships between income inequalities and happiness or subjective well-being are considered, some authors find a negative relationship between income inequalities and happiness and explain it with the perceived unfairness in the distribution and mistrust. Moreover, the findings also show that with full equality in income distribution, happiness is at a low level because of the subjective feeling that full equality is unfair. Tavor et al. analyzed different levels of inequalities relations with happiness. They observed that human beings are aware of the varying contributions of individuals that should be rewarded with differing incomes. That is why very low inequalities had neither positive nor negative impact on happiness. On the contrary, high Gini coefficient significantly and negatively affected happiness [19, 20]. At the same time, countries with the most stable and clear patterns in income distribution (measured by Gini coefficient) have more obvious successes in social and economic spheres, including human development level [21, 22]. Often these differences cause large-scale migration [23, 24], disparities in regional development [25, 26], especially being by influence of connected factors, such as political climate [27]. Also, time may have an impact on this relation. Short-run increases in inequality, rather than long run persistent high level of inequality, make people less satisfied with their life [28]. What is more, Europeans’ subjective well-being is more affected than Americans by inequalities because of lower perceived possibility of social mobility (where individual effort can move people up the income ladder) in European countries [29]. With the possibility of social mobility, the observation of others increasing their income may increase expectations about the future and make people happier. Therefore, people may accept inequality if it signals social mobility [30]. In this perspective, the relationship between education and income inequalities may play an important role. Because high-income families invest more heavily in their children’s early education, their children receive more schooling than children with the same innate ability who are from low-income families. The gap between children from families in the top income quantile and children from families in the bottom income quantile increases at the end of compulsory education and further increases at the end of higher education [31].

Recent empirical observation supports the hypothesis that higher inequality does have a negative impact on individual well-being levels because of social comparisons, so even if absolute income increases, the relative position in the income distribution could worsen [7]. Previous findings reveal the evidence of strong associations between material wealth and life evaluation at both individual and nation levels [32]. However, material dimension can have a weak relationship with subjective well-being in transition countries, because people are focusing on other dimensions of well-being to fulfill other needs. Nevertheless, when there is high-income inequality, comparisons may create negative feelings not only for the poor but also for the wealthy [9, 33]. What is more, the people’s material standard of living improves with growing income but they adapt to better conditions and raise their evaluative standards. People do not become happier because their rising income/wealth may not keep up with their increasing material desires [34, 35]. Subjective well-being may also have positive associations with unequal distribution of income. Income inequality has more positive effects on individuals who are relatively better and have moderate perceptions of poverty being caused by unfairness than those that perceive the income generation process unfair. Therefore, reducing social comparisons and increasing perceptions of fairness, as well as beliefs about hard work leading to success, are suggested to increase well-being. Abilities to cope with life and economic perspective for improvements are improved by more equal distribution, which on other hand, increases happiness [36]. An individual’s expression of social trust may be an important factor affecting the positive relationships between income inequalities and well-being and can explain the relation that more unequal countries report higher subjective well-being [37].

The evidence that large-income differences have damaging health and social consequences is strong and, in most countries, inequality is increasing. The indirect impact was identified by Angus Deaton, who identified the health gradient—richer people are healthier, more effective, and live better [38]. Narrowing the income gap will improve not only the health but also the well-being of populations [7, 39].

Environment degradation could be considered as a channel through which income distribution affects a population’s health. Income inequalities negatively affect environmental quality, and that environment degradation worsens a population’s health [40]. However, relationship between income inequality and per capita emissions depends on the level of income. For low- and middle-income economies, higher-income inequality is associated with lower carbon emissions, whereas in upper middle-income and high-income economies, higher-income inequality increases per capita emissions [41].

In conclusion, there is no consensus about the direction and strength of the relationships between income inequalities and well-being in the literature. However, general findings suggest that although the impacts of income inequality differ across various dimensions of well-being, reducing inequality will generally help improve the well-being of a society [42]. What may have had affected the inconclusiveness of the finding was the different measures of well-being and income inequalities, countries belonging to different welfare regimes. What is more, there is a lack of the analysis for the group of postsocialist countries. Therefore, a broader understanding of inequalities concerns their associations not only with regard to their incomes but also with respect to other indicators of well-being and involvement in society. The ultimate question: Is inequality related to the level of well-being in its particular dimensions in societies of CEE countries?

## Methods and data

### Well-being and income inequalities—Data

The terms well-being, welfare, and quality of life are frequently used synonymously [10]. In our article, we use the term “well-being” in the sense of overall condition (standard of living) that consists of 5 dimensions (material dimension, health dimension, education dimension, natural environment, and happiness). However, the different meaning of well-being, such as evaluative (overall life evaluation measured by the best possible life question) and hedonic (daily experience, as measured by smiling yesterday), may also be considered [12].

Table 1 offers the dimensions of well-being and indicators used in five of its analyzed dimensions (material, health, education, natural environment, and happiness).

**Table 1:** The description and sources of data concerning dimensions of well-being.

Variable	Dimension	Description	Source
**MD**	Material dimension	Adjusted gross disposable income of households per capita (PPS) per inhabitant	Eurostat
**HD**	Health dimension	Life expectancy at birth	World Bank
**EDU**	Education dimension	Primary completion rate	World Bank
**NED**	Natural environment dimension	CO_2_ emission per capita	International Energy Agency
**HS**	Happiness	Happiness index	World Database of Happiness

Source: own evaluation on the basis of Eurostat, World Bank, World Database of Happiness, International Energy Agency.

**Fig 1:**
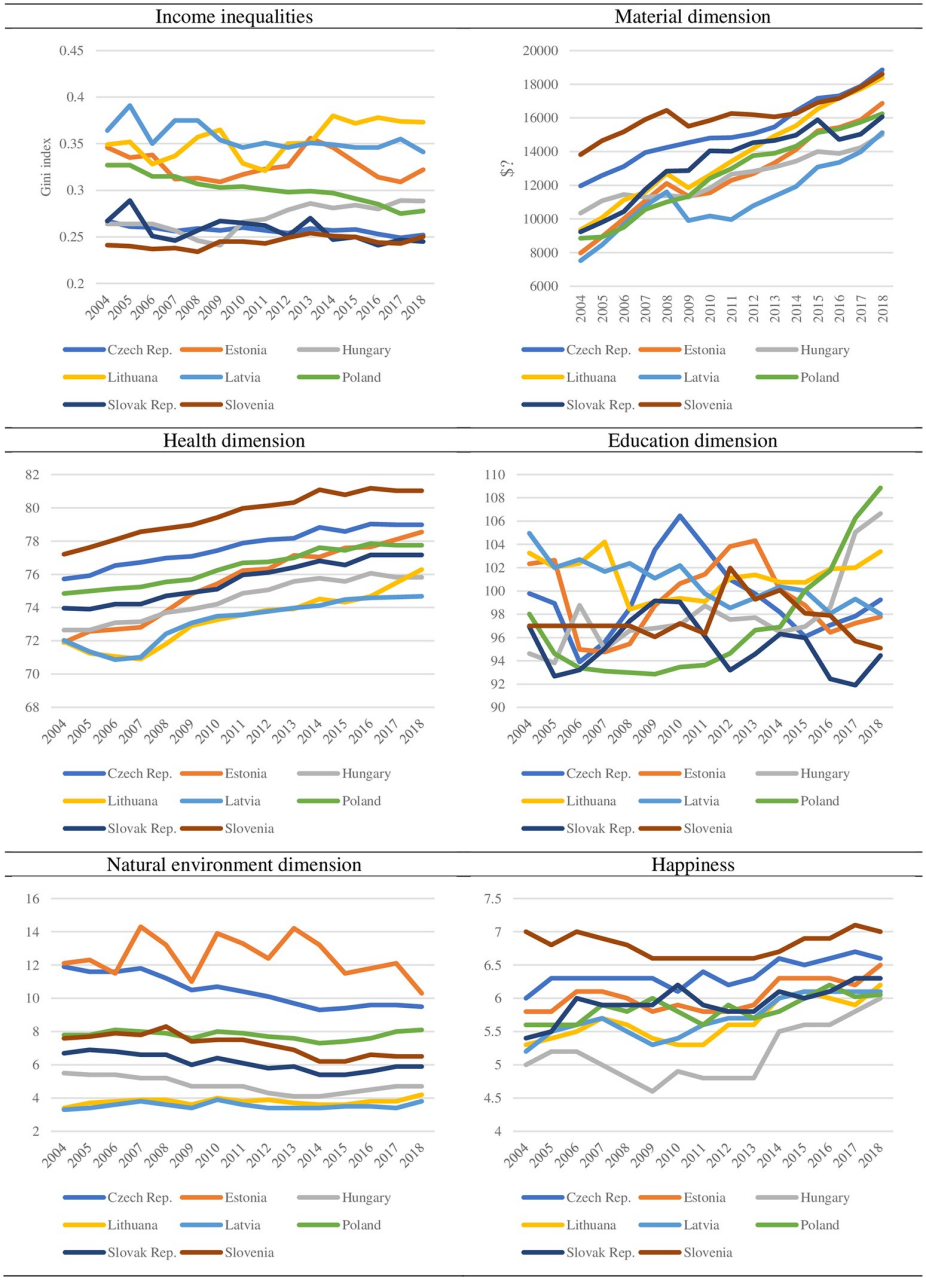
Income inequalities and five dimensions of well-being in selected CEE countries. Source: Own evaluation on the basis of OECD, Eurostat, World Bank, World Database of Happiness, International Energy Agency.

Material well-being dimension is described by adjusted gross disposable income of households per capita (PPS) per inhabitant [43]. The gap between the countries decreased between 2004 and 2018. In 2004, Latvia was identified as the lowest level of gross disposable income per capita (7514 PPS) and Slovenia as the highest (13819 PPS). In 2018, Hungary was identified as the lowest level in material dimension of well-being (15010 PPS) and Czech Republic as the highest (18857 PPS) at the same time. The highest average growth rate of disposable income per capita was observed in Estonia (5,6%) and lowest in Slovenia (2,2%) between 2004 and 2018. However, this dimension improved in all CEE countries to a relatively high extent, indicating the catching up to the European Union (Fig 1).

Life expectancy has increased, however, to the lower extent than in the rest of the European Union countries, where it reached the threshold level of 80 years or longer in 2004 in most countries. In the year of the accession to the EU, the average life expectancy in CEE countries was 74 years, with the lowest in Estonia (72 years) and the highest in Slovenia (77 years). In 2018, this dimension of well-being improved but was more diversified. The highest life expectancy was also in Slovenia (81 years—the only country with life expectancy older than 80 years) and lowest in Latvia (74 years) (Fig 1).

When analyzing education dimension, the CEE countries have undertaken a special effort to raise educational level and are among the countries with the highest share of people that have attained at least secondary level [43]. When primary completion rate is considered, the highest possible levels are reached by all the CEE countries, with the best situation in Poland and worst in Slovak Republic in 2018 [44]. However, the results in these dimensions were changing in the different directions and were diversified in 2018 in CEE countries.

The achievements in natural environment dimension remained relatively stable across CEE countries between 2004 and 2018. However, the improvement would be interpreted as the decrease in the CO_2_ emissions per capita. The decreasing trend was observed in all CEE countries except Poland, Latvia, and Lithuania. What is more, CEE countries reach very different levels of CO_2_ emissions per capita ranging from above 10 in Estonia to less than 4 in Latvia in 2018. However, the gap between country with highest emission of CO_2_ per capita (12.3 in Estonia) and lowest (3.3 in Latvia) in comparison to 2004 decreased (Fig 1).

When subjective well-being is evaluated on the basis of happiness index, all the CEE countries are below the EU- average [10, 45]. What is interesting is that most of these countries theoretically should be characterized with a higher level of life satisfaction if a cross-country regression of life satisfaction on GDP per capita (adjusted for PPP) was performed [12]. The citizens of CEE countries were, on average, less satisfied with their lives than are those of other countries with comparable level of income during the transformation process [12]. The positive contribution to life satisfaction of improved material living standards was outweighed by losses in employment security, health, and child care might be the reason [33]. Happiness dimension of well-being improved, on average, from the 5.7 level in 2004 to 6.5 level in 2018. Both at the moment of joining the EU and 2018, the lowest level of happiness was observed in Hungary and the highest in Slovenia. However, the distance between the least happy society and the most happy society in the CEE countries decreased (Fig 1).

Income inequalities calculated on the basis of the disposable incomes (Gini coefficient after taxes and transfers, OECD IDD database) were at different levels across CEE countries between the 2004 and 2018 period. However, recent inequality research provides convincing evidence that survey data seem to be a less credible source of information about the levels of income than data derived from administrative sources, for instance, individual tax returns and aggregated income tax statistics [46]. Based on the Gini coefficient, in the whole period, income distribution was the most unequal in Baltic countries. There were the highest levels of income inequalities in Lithuania, Latvia, and Estonia in 2004 as well as in 2018. The peak in income inequalities among the CEE countries in the analyzed period was reached in 2015 in Latvia (0.39). On the other side, Slovenia was the country with the lowest level of income inequalities in whole period (0.24). The highest decrease in income inequalities was identified in Poland (from level 0.33 in 2004 to level 0.28 in 2018). Income inequalities increased in Lithuania (from 0.35 in 2004 to 0.37 in 2018) and Hungary (from 0.27 in 2004 to 0.29 in 2018) (Fig 1).

### Panel ARDL model and PMG estimator

With the aim of examining the relationship between income inequalities (*IEE*_it_) and five dimensions of well-being (*MD*_it_, *HD*_it_, *EDU*_it_, *NED*_it_, *HS*_it_), this study adopts the panel dynamic methods. We consider the panel autoregressive distributive lag model (panel ARDL(*q*, *p*, *p*, *…*, *p*)), and the theoretical form of the model can be written as follows [47]:

$$y_{it}=\alpha^{'}+\sum_{j=1}^{q}\alpha_{ij}y_{i,t-j}+\sum_{j=1}^{p}\beta_{ij}x_{i,t-j}+\varepsilon_{i,t}$$ (1)

where *y*_*it*_ represents the dependent variable, *x*_*i*,*t*−*j*_ is a vector of explanatory variables, *α*_*ij*_, *β*_*ij*_, denotes the parameters of the explanatory variables, *i* = 1, 2, 3, …., *N* represents the individual countries, and *t* = 1, 2, 3, …, *T* are the periods, *q*, *p* denotes the lag order on the variables, and *ε*_*i*,*t*_ represents the white noise error terms. In this study, six models are formulated in the form of panel ARDL (*q*, *p*, *p*, *…*, *p*) model and takes the following forms:

*Equation for income inequalities* (IEEit)

$$\Delta {IEE}_{it}=c_{1}+\phi_{1,i}\left[{IEE}_{i,t-1}-\vartheta_{1,i}^{'}\left({MD}_{i,t-1}+{HD}_{i,t-1}+{EDU}_{i,t-1}+{NED}_{i,t-1}+{HS}_{i,t-1}\right)\right]+\sum_{j=1}^{q-1}\beta_{ij}{\Delta IEE}_{i,t-j}+\sum_{j=0}^{p-1}\alpha_{ij}{\Delta MD}_{i,t-j}+\sum_{j=0}^{p-1}\gamma_{ij}{\Delta HD}_{i,t-j}+\sum_{j=0}^{p-1}\delta_{ij}{\Delta EDU}_{i,t-j}+\sum_{j=0}^{p-1}\varphi_{ij}{\Delta NED}_{i,t-j}\sum_{j=0}^{p-1}\psi_{ij}{\Delta HS}_{i,t-j}+\varepsilon_{it}$$ (1.1)

*Equation for material dimension* (MDit)

$$\Delta {MD}_{it}=c_{2}+\phi_{2,i}\left[{MD}_{i,t-1}-\vartheta_{1,i}^{'}\left({IEE}_{i,t-1}+{HD}_{i,t-1}+{EDU}_{i,t-1}+{NED}_{i,t-1}+{HS}_{i,t-1}\right)\right]+\sum_{j=1}^{q-1}\beta_{ij}{\Delta IEE}_{i,t-j}+\sum_{j=0}^{p-1}\alpha_{ij}{\Delta MD}_{i,t-j}+\sum_{j=0}^{p-1}\gamma_{ij}{\Delta HD}_{i,t-j}+\sum_{j=0}^{p-1}\delta_{ij}{\Delta EDU}_{i,t-j}+\sum_{j=0}^{p-1}\varphi_{ij}{\Delta NED}_{i,t-j}\sum_{j=0}^{p-1}\psi_{ij}{\Delta HS}_{i,t-j}+\varepsilon_{it}$$ (1.2)

*Equation for health dimension* (HDit)

$$\Delta {HD}_{it}=c_{3}+\phi_{3,i}\left[{HD}_{i,t-1}-\vartheta_{1,i}^{'}\left({{IEE}_{i,t-1}+MD}_{i,t-1}+{EDU}_{i,t-1}+{NED}_{i,t-1}+{HS}_{i,t-1}\right)\right]+\sum_{j=1}^{q-1}\beta_{ij}{\Delta IEE}_{i,t-j}+\sum_{j=0}^{p-1}\alpha_{ij}{\Delta MD}_{i,t-j}+\sum_{j=0}^{p-1}\gamma_{ij}{\Delta HD}_{i,t-j}+\sum_{j=0}^{p-1}\delta_{ij}{\Delta EDU}_{i,t-j}+\sum_{j=0}^{p-1}\varphi_{ij}{\Delta NED}_{i,t-j}\sum_{j=0}^{p-1}\psi_{ij}{\Delta HS}_{i,t-j}+\varepsilon_{it}$$ (1.3)

*Equation for education dimension* (EDUit)

$$\Delta {EDU}_{it}=c_{4}+\phi_{4,i}\left[{EDU}_{i,t-1}-\vartheta_{1,i}^{'}\left({IEE}_{i,t-1}+{MD}_{i,t-1}+{HD}_{i,t-1}+{NED}_{i,t-1}+{HS}_{i,t-1}\right)\right]+\sum_{j=1}^{q-1}\beta_{ij}{\Delta IEE}_{i,t-j}+\sum_{j=0}^{p-1}\alpha_{ij}{\Delta MD}_{i,t-j}+\sum_{j=0}^{p-1}\gamma_{ij}{\Delta HD}_{i,t-j}+\sum_{j=0}^{p-1}\delta_{ij}{\Delta EDU}_{i,t-j}+\sum_{j=0}^{p-1}\varphi_{ij}{\Delta NED}_{i,t-j}\sum_{j=0}^{p-1}\psi_{ij}{\Delta HS}_{i,t-j}+\varepsilon_{it}$$ (1.4)

Equation for natural environment dimension (NEDit)

$$\Delta {NED}_{it}=c_{5}+\phi_{5,i}\left[{NED}_{i,t-1}-\vartheta_{1,i}^{'}\left({{IEE}_{i,t-1}+MD}_{i,t-1}+{HD}_{i,t-1}+{EDU}_{i,t-1}+{HS}_{i,t-1}\right)\right]+\sum_{j=1}^{q-1}\beta_{ij}{\Delta IEE}_{i,t-j}+\sum_{j=0}^{p-1}\alpha_{ij}{\Delta MD}_{i,t-j}+\sum_{j=0}^{p-1}\gamma_{ij}{\Delta HD}_{i,t-j}+\sum_{j=0}^{p-1}\delta_{ij}{\Delta EDU}_{i,t-j}+\sum_{j=0}^{p-1}\varphi_{ij}{\Delta NED}_{i,t-j}\sum_{j=0}^{p-1}\psi_{ij}{\Delta HS}_{i,t-j}+\varepsilon_{it}$$ (1.5)

Equation for happiness (HSit)

$$\Delta {HS}_{it}=c_{6}+\phi_{6,i}\left[{HS}_{i,t-1}-\vartheta_{1,i}^{'}\left({{IEE}_{i,t-1}+MD}_{i,t-1}+{HD}_{i,t-1}+{EDU}_{i,t-1}+{NED}_{i,t-1}\right)\right]+\sum_{j=1}^{q-1}\beta_{ij}{\Delta IEE}_{i,t-j}+\sum_{j=0}^{p-1}\alpha_{ij}{\Delta MD}_{i,t-j}+\sum_{j=0}^{p-1}\gamma_{ij}{\Delta HD}_{i,t-j}+\sum_{j=0}^{p-1}\delta_{ij}{\Delta EDU}_{i,t-j}+\sum_{j=0}^{p-1}\varphi_{ij}{\Delta NED}_{i,t-j}\sum_{j=0}^{p-1}\psi_{ij}{\Delta HS}_{i,t-j}+\varepsilon_{it}$$ (1.6)

where *c* is constant, *ε*_*it*_ are the residuals, and the explanatory variables were defined as a logarithmic levels and first differences of logarithmic-dependent variables. *ϕ*_·,*i*_ are the error correction terms in each equation. They are expected to be negative (value between [−1; 0]) so as to show evidence of long-run relationship. The value of error correction term shows the speed of adjustment to the long-term equilibrium. *β*_*ij*_, *α*_*ij*_, *γ*_*ij*_, *δ*_*ij*_, *φ*_*ij*_, *ψ*_*ij*_ are the short-run parameters that present the short-term relationship between variables.

To calculate the parameters of all the equations, the *pooled mean group* estimator (*PMG*) was used. The method was developed by [47, 48] and is increasingly popular in research. The method is designed for significant and known problems related to panel data modeling, which are dependence of left hand–side variable on its own past realization, heteroscedasticity, fixed individual effects, and autocorrelation. The PMG estimator controls the long-run parameters to be constant across individual country groups while allowing the intercept, short-term estimates and variance of the errors to differ across the group [47]. The reason to expect the long-run equilibrium relationships between variables are constant across individuals is related to the common technologies influencing all groups in a similar way, arbitrage condition, or because of budget and solvency constraints. The ARDL model recently has been widely used because of the fact that it can be used regardless of whether the variables are *I*(0) or *I*(1) and can be used to generate the short-run and long-run estimates simultaneously. Also, the PMG estimator is suitable when N (number of individual groups) and T (time periods) are both large or both small. Authors considered the use of estimator for large panel (N = 24 and T = 32), as well as for small panel (N = 10 and T = 17) [47].

The use of PMG estimator is related to transforming all variables by differencing, that is, Δ*y*_*i*,*t*_ = *y*_*i*,*t*_ − *y*_*i*,*t*−1_ and Δ*x*_*i*,*t*_ = *x*_*i*,*t*_ − *x*_*i*,*t*−1_. Transformation of all variables and using the first differences of this variables in the study is also associated with the unit root analysis. For this purpose, we used a well-known method of panel unit root test. We examined the stationarity of all variables based on LLC test [49], IPS test [50], and Fisher type unit root tests [51]. For more information regarding panel unit root analysis, see the indicated literature. Also, all variables are used with their natural logarithms to reduce heteroscedasticity and to obtain the growth rate of the relevant variables by their differenced logarithms.

## Empirical results

### Panel unit root tests and properties of empirical models

We examine the relationship among income inequalities, material dimension, health dimension, education dimension, environment dimension, and happiness by using panel ARDL models.

Before the models for income inequalities and other five dimensions of well-being were built, unit root tests were performed. We used two types of tests, the first assumes the common unit root process for all individuals (Levin, Lin, and Chu test), the second assumes individual unit root process for all individuals (Im, Pesaran, Shin test, and Fisher- type tests). The results of panel unit root tests are presented in Table 2.

**Table 2:** Results of panel unit root test.

Dimension Unit root test	Income inequalities (*IEE*)		Material dimension (*MD)*	
	Stats for levels	Stats for differences	Stats for levels	Stats for differences
	Intercept and trend	Intercept	Intercept and trend	Intercept
*H*_0_: Unit root (assumes common unit root process)				
Levin, Lin & Chu (t*)	−2.019 **	−3.856 ***	0.00030	−3.829 ***
*H*_0_: Unit root (assumes individual unit root process)				
Im, Pesaran and Shin (W-stat)	−1.03056	−4.00844 ***	0.80490	−3.18338 ***
ADF—Fisher (*χ*^2^)	19.9328	45.1936 ***	8.95587	37.1009 ***
PP—Fisher *χ*^2^	28.0247 **	102.732 ***	8.39576	56.1883 ***
Decision	*I*(1)		*I*(1)	
Dimension	Education dimension (*EDU*)		Health dimension (*HD*)	
*H*_0_: Unit root (assumes common unit root process)				
Levin, Lin & Chu t*	−0.45291	−2.34180 ***	2.77213	−1.34143 *
*H*_0_: Unit root (assumes individual unit root process)				
Im, Pesaran and Shin W-stat	0.70177	−2.72008 ***	2.58990	−1.93087 **
ADF—Fisher χ^2^	16.7421	34.6029 ***	8.52401	26.6761 **
PP—Fisher χ^2^	17.4354	74.1164 ***	9.77948	77.5888 ***
Decision	*I*(1)		*I*(1)	
Dimension	Natural environment dimension (*NED*)		Happiness (*HS*)	
*H*_0_: Unit root (assumes common unit root process)				
Levin, Lin & Chu t*	1.33352	−5.15465 ***	−0.92162	−4.21713 ***
*H*_0_: Unit root (assumes individual unit root process)				
Im, Pesaran and Shin W-stat	0.83788	−3.94105 ***	0.59508	−3.51025 ***
ADF—Fisher χ^2^	10.9465	44.8119 ***	12.2390	40.9377 ***
PP—Fisher χ^2^	15.0226	81.1472 ***	14.1766	77.5706 ***
Decision	*I*(1)		*I*(1)	

Source: own evaluation.

The results of the unit root tests for variables with individual intercept or with individual intercept and trends shows that all well-being dimensions are stationary in first differences, but not stationary in levels (see Table 2). Levin, Lin, and Chu test, as well as Im, Pesaran, and Shin test and Fisher type (ADF and PP) tests does not reject the null hypothesis of unit root for levels, but the null hypothesis of second-order integration—*I*(2) for five dimensions of well-being are rejected when the first differences of variables are considered (see Table 2).

Only for income inequalities, the Levin, Lin, and Chu test shows that there is no common unit root for all countries included in the research. This means that the income inequalities, at least in one country, are stationary. Then, we use the unit root tests (IPS test and Fisher type test), which assumes an individual unit root in *IEE*_it_ for analyzed countries. Two of three tests (IPS test and ADF test) show that the income inequalities in individual cross-sections are integrated are of the first order (see Table 2). However, the PP-Fisher test rejects the null of individual unit root for *IEE*_it_. Because of the construction of the alternative hypothesis, the rejection of the null hypothesis does not mean that there is no unit root in the panel, but the null hypothesis has been rejected for some individuals. Hence, based on the results of Im, Pesaran, Shin test and ADF Fisher-type test, we find that the income inequalities, as well as other variables, are non-stationary in levels and stationary in first differences. In a further study, we use the first differences of all the variables.

Based on the annual data for eight CEE economies, the parameters of panel ARDL(*q*, *p*, *p*, *p*, *p*, *p*) model were estimated, and the statistical properties of the residuals were assessed. The estimator we used is a popular method for estimating dynamic panels with different short-run coefficients and error variances across individuals. For built ARDL models, the JB test for the normality of the residuals distribution was calculated. The results of tests are presented in Table 3.

**Table 3:** Residual diagnostic of empirical models.

Test	Eqs (1.1)–(1.6)					
	IIE_it_	MD_it_	HD_it_	EDU_it_	NED_it_	HS_it_
Lag length	(1,1,1,1,1,1)	(1,1,1,1,1,1)	(1,1,1,1,1,1)	(1,1,1,1,1,1)	(1,1,1,1,1,1)	(1,1,1,1,1,1)
Normality test (JB):	57.38***	14.53***	2.72	7.89***	263.7***	5.04*
Skewness	0.886	0.56	0.22	0.09	0.22	0.34
Kurtosis	6.025 ***	4,36 **	3,62	4,28 **	10.5 ***	3,78 *
Number of countries	8	8	8	8	8	8
Observations	120	120	120	120	120	120

Source: own evaluation.

Residual diagnostics in all equations show that all instruments used in panel ARDL models are valid. However, based on normality test, we concluded that the residuals of the four equations does not have a normal distribution. The *p* value for *JB* statistics is smaller than the significance value (α = 0.05) for IEE, MD, EDU, and NED equations. The lack of normality of the distribution is because of the presence of significant kurtosis of the distribution (kurtosis coefficients in all equations are significantly larger than 3) rather than the insignificant asymmetry of the distribution [52], which is less negative to the results (see Table 3).

### Preliminary analysis of the relationships among income inequalities and dimensions of well-being—Correlation matrix analysis

The correlation matrix among income inequalities and well-being dimensions (material, health, education, environment, and happiness) is presented in Table 4.

**Table 4:** Correlation matrix between variables.

Variables	IIE_it_	MD_it_	HD_it_	EDU_it_	NED_it_	HS_it_
IIE_it_	**1**	− **	− ***	+ **	− *	− **
MD_it_		**1**	+ ***	+	**+**	+ ***
HD_it_			**1**	−	+ **	+ ***
EDU_it_				**1**	−	−
NED_it_					**1**	+ **
HS_it_						**1**

Note:

***, strong <0,6; 1,0> and significant correlation;

**, moderate <0,3; 0,6) and significant correlation,

*, weak (0,3; 0,0> and significant correlation; other, unsignificant correlation;

^**+**^, positive correlation;

^−^, negative correlation.

Source: own evaluation.

The preliminary correlation analysis shows that four of five analyzed dimensions of well-being (material, health, environmental, and happiness) are negatively and significantly correlated with income inequality, where the correlation coefficient for HD_it_ indicates strong dependency. For MD_it_ and HS_it_, shows a moderate relationship; and for NED_it_, it means weak dependency. The negative signs of correlation coefficient means that certain dimensions can reduce income inequalities in CEE countries (see Table 4). The correlation coefficient between education dimension (*EDU*_*it*_) and income inequalities has a positive value, which suggests the positive and moderate relations between access to education and inequality of income.

The correlation relationship between material dimension (MD_it_) and health dimension (HD_it_) is strong, positive, and significant, which means the gross disposable income of households per capita can positively affect life expectancy at birth. Correlation relationship between both material dimension (MD_it_) and happiness (HS_it_), as well as happiness (HS_it_) and health dimension (HD_it_), are strong, positive, and significant. It means that both dimensions are significant for the increase of happiness (see Table 4). The next step was to build ARDL models describing the short-run and long-run relationships among income inequalities and well-being dimensions.

### Long-run and short-run analysis of relationships between income inequalities and dimensions of well-being—Results from annual data for CEE countries

Panel ARDL model and PMG estimator is appropriate whether the underlying regressors exhibit I(0) or I(1) and both *N* (number of individuals) and *T* (time span) are relatively small [47]. To identify the relationships among income inequalities, material dimension, health dimension, education dimension, environmental dimension, and happiness, we have applied the Student *t* test, wherein the *p* values were marked and presented in Table 5.

**Table 5:** PMG estimation results for panel of CEE countries (panel ARDL models)—Annual data.

	IIE_it_	MD_it_	HD_it_	EDU_it_	NED_it_	HS_it_
	Coefficient	Coefficient	Coefficient	Coefficient	Coefficient	Coefficient
Panel A—Long Run Equation						
log(IIE_it_)	—	0,596 ***	0,058 ***	0,453 ***	−1,204 ***	0,267 **
log(MD_it_)	0,437 ***	—	0,171 ***	-0,204 ***	−0,666 ***	0,272 ***
log(HD_it_)	−1,013 ***	4,403 ***	—	0,318 **	4,404 ***	0,248
log(EDU_it_)	1,795 ***	−1,386 ***	−0,059 ***	—	0,733 ***	−0,225 ***
log(NED_it_)	−0,420 ***	0,359 ***	−0,005	0,177 ***	—	0,113 **
log(HS_it_)	−1,076 ***	2,275 ***	−0,281 ***	0,498 ***	1,618 ***	—
Panel B—Short Run Equation						
*ECM*_it-1_	−0,397 *	−0,306 **	−0,360 ***	−0,308	−0,357 **	−0,554 ***
Δ log(IIE_it_)	—	0,160	−0,050 *	−0,035	−0,132	−0,248 *
Δ log(MD_it_)	−0,035	—	−0,044 **	−0,092	0,719 ***	0,136 ***
Δ log(HD_it_)	−0,639	−1,570	—	0,212	−0,673	−2,789 ***
Δ log(EDU_it_)	−0,686 *	0,458	−0,017	—	0,114	0,101
Δ log(NED_it_)	0,055	0,087	0,002	−0,020	—	0,021
Δ log(HS_it_)	0,146	−0,349	0,028	−0,046	−0,644 **	—
C	−2,616 *	−2,216 **	1,284 ***	1,399	−6,637 **	−0,393

Source: own evaluation.

The results of the long-term and short-term coefficients estimation in Eqs (1.1) to (1.6) are presented in Table 5.

Most of the point estimates of long-run parameters in selected equations are consistent with economic theory. Some of the long-run coefficients are important for the achievement of the research aim, and therefore, only selected parameters will be interpreted. First of all, the long-run coefficient shows the significant long-run interdependencies between variables. Based on equation for income inequalities, we can say that health dimension (HD_it_) and natural environment dimension (NED_it_) reduce the income inequalities in CEE countries. An increase by 1% in health dimension and environmental dimension cause the decrease of income inequalities by 1.013% and 0.42%, respectively. Also, the long-run coefficient for happiness in income inequalities equation shows that the increases in the sense of happiness (HS_it_) by 1% cause the decreases in income inequalities (IIE_it_) by 1.076%. The other two dimensions (material and education) seem to contribute to an increase in income inequality in the long run. An increase in those two dimensions by 1% tends to increase in income inequalities by 0.437% and 1.795%, respectively (Table 5).

Higher-income inequalities cannot be confused with poverty. What is more, the richer the society, the more is spent to protect the most vulnerable groups of population [53]. Therefore, in the period, after the accession to European Union, the increase in income inequalities was positively related to the increase in household disposable income per capita in CEE countries. Education increases income inequalities in the long run, because the more people are qualified, the better they are educated, the more they can earn. So, improvements in the education dimension contribute to the increase in income inequalities in the long run. The long run negative relationship between the health dimension and income inequalities proves that the findings observed in the literature [7], as well as negative relationship between happiness and income inequalities [19, 20].

Based on equations estimated for the dimension of well-being, the positive and significant impact of income inequalities on material dimension, health dimension, education dimension, and happiness was identified. An increase in income inequalities by 1% creates an average increase in material dimension, health, education, and happiness dimensions by 0.596%, 0.058%, 0.453%, and 0.267%, respectively. Increase of income inequalities contributed to the decrease of natural environment dimension only. When income inequalities are considered as the natural consequence of economic development in the long run [53], it does not seem controversial that the increase in income inequalities contributed to the increase in material dimension, health dimension, education, and happiness (Table 5).

As previously stated, the panel ARDL model can be used to account for long-run and short-run relationships, as well as the speed of adjustment to the long-run equilibrium (the error correction term). The Error Correction Model has a significant negative sign for the error correction term, which implies that the model converges to a long-run relationship. In the equation for *income inequalities* (IEEit), the average point estimate of error correction (*ϕ*_*i*_ = −0.397) shows that about 39.7% of the deviation from equilibrium in CEE economies is corrected in the next year. In the equations describing the dimensions of well-being, the parameters of adjustment to long-term equilibrium also assume values consistent with the theory (negative in range 0 to −1.0) and shows that the short-term fluctuations in the dimensions of well-being are able to correct the deviations from the long-term path by nearly 30.6% to 55.4% in the next year (the range of speed of adjustment in Eqs (1.2) to (1.6) is −0.306 to −0.554) (see Table 5).

**Fig 2:**
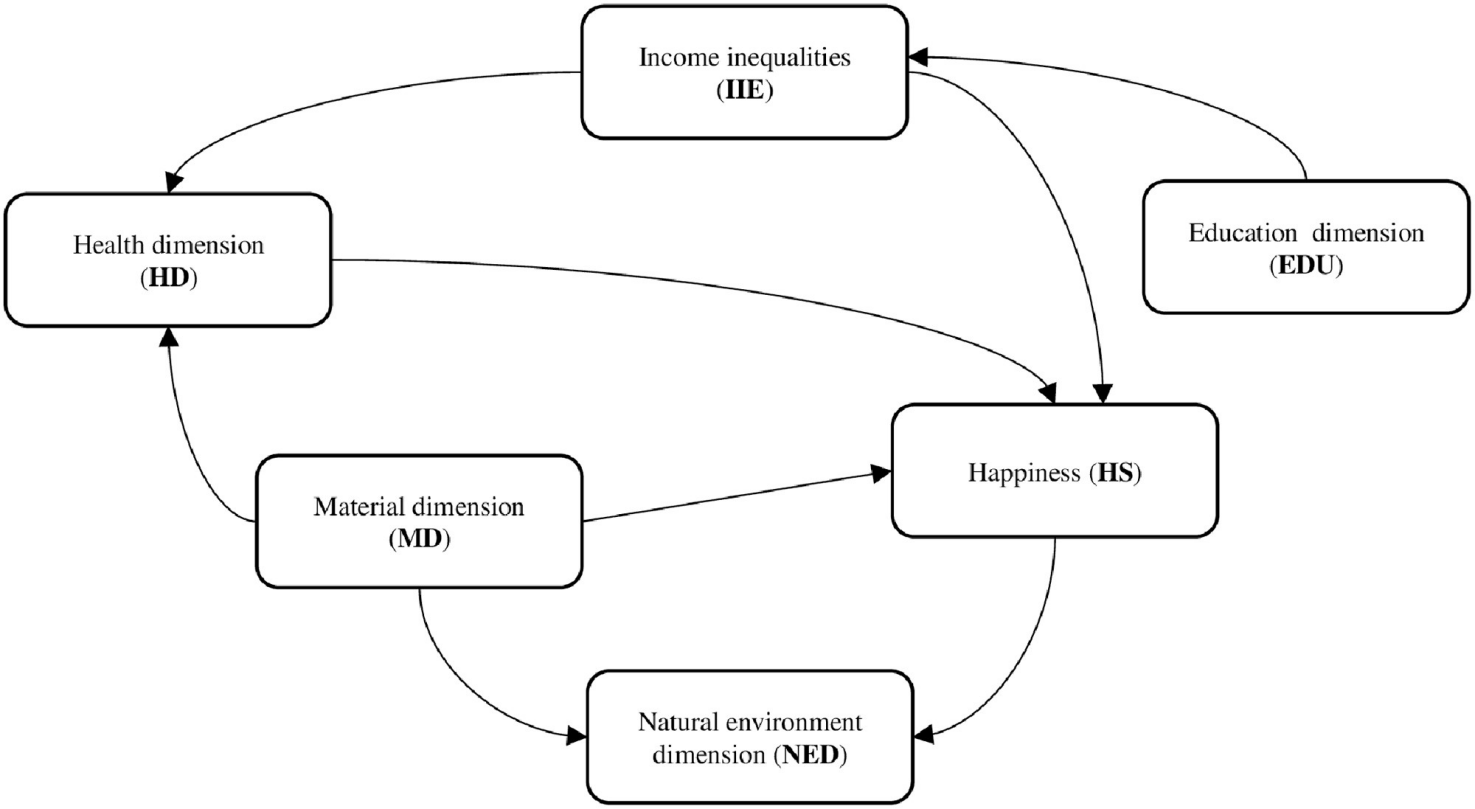
Scheme 1. Short-run links among income inequalities and dimensions of well-being—annual data. Source: own evaluation based on Table 5.

Given the above results of long-run coefficients analysis, it was identified that the income inequalities in long term were shaped by all dimensions of well-being, such as material dimension, health dimension, education, natural environment, and the subjective dimension of well-being—happiness. However, the short-run coefficient analysis shows that only education dimension of well-being bears the burden of short-run adjustment to restore long-run equilibrium after a shock to the system. Here, we can say that the short-run increase in education dimension by 1% tends to decrease the inequalities of income by 0.686% in CEE economies. Negative and significant short-run relationship, which was identified between education dimension and income inequalities, suggests that the education dimension can reduce the income inequalities in short term. The impact of short-run stabilization and re-distributional policies as increased expenditures on training and education may have played an important role in the result that more people have the access and money to learn for longer periods. However, based on short-run equations for different dimension of well-being, the negative and significant impact of income inequalities was identified on health dimension and happiness. The average short-run impact of variables in all 8 CEE countries to income inequalities and other dimensions of well-being is presented in Table 5, panel B. Based on short-run relations among all variables, the scheme of links were built and presented below in Fig 2.

The results of short-term relationships, based on the panel ARDL models, show that there is short-run unidirectional relationship running from income inequalities to health dimension (*IEE*_*it*_ → *HD*_*it*_), as well from income inequalities to happiness (*IEE*_*it*_ → *HS*_*it*_). Also, there are linkages between education dimension and income inequalities (*EDU*_*it*_ → *IEE*_*it*_). The material dimension is a cause for health dimension (*MD*_*it*_ → *HD*_*it*_), but it also determines the feeling of happiness (*MD*_*it*_ → *HS*_*it*_) and the environmental dimension (*MD*_*it*_ → *NED*_*it*_). Also, there is a short-run unidirectional relationship running from happiness dimension (HS_it_) to natural environment dimension (NED_it_) (Table 5; Scheme 1 in Fig 2).

The short-run coefficient analysis shows also that only education dimension of well-being bears the burden of short-run adjustment to restore long-run equilibrium after a shock to the system. Also, it is worth noticing that increases in income inequalities in the short term can reduce the health dimension and subjective well-being (happiness), respectively, by 0.05% and 0.248% (Table 5). The impact of income inequality on the health dimension is negligible, but it is more important for the feeling of happiness.

Based on Scheme 1 (Fig 2), it can be observed that the only dimension that shapes the income inequalities in the short term is the education dimension; however, through the income inequalities, education affects the other dimensions of well-being indirectly (health and happiness). Income inequalities can be interpreted as a factor that shapes the economic process in which individuals make decisions affecting health and belonging to various social classes and achieving happiness. However, it is the unequal distribution of income that depends on education dimension. The short-run relationship running from material dimension to subjective well-being (happiness), as well as health dimension and natural environment, shows that the disposable income per capita plays important role in shaping happiness both directly and indirectly through health. What is more material dimension was directly and indirectly through happiness linked to natural environment in the short run. In natural environment degradation, the climate change is shaped by material dimension; however, it is also affected by happiness. The negative short-run impact of subjective well-being to CO_2_ emissions revealed an interesting relationship because society, which achieves higher level of life satisfaction, may protect natural environment to a higher extent.

## Checking the results of the analysis for the larger T—Re-estimation of panel ARDL model

In this study, the long-term and short-term relationships between variables were computed for small number of individuals (N = 8, eight CEE countries) and small number of periods (T = 15). That is why the panel ARDL model for larger data panel sample was also used to study the relationships between inequalities and well-being dimensions. The additional models were built to check and compare the results obtained from the models for relatively small N and T. Therefore, we interpolated annual data to quarterly frequency by using the Denton-Cholette method [54] to obtain the larger sample. The new sample period is from 2004 (Q1) to 2018 (Q4). All variables are used with their natural logarithms to reduce heteroscedasticity and to obtain the growth rate of the relevant variables by their differenced logarithms.

The findings from the PMG estimation for quarterly panel data for CEE countries are available upon request from the authors. However, some similarities and differences with the model on the annual data are presented below. First, the ARDL models with 3 lags for dependent variable and 4 lags for explanatory variables were fitted to most of the cases (ARDL (*3*, *4*, *4*, *4*, *4*, *4*)). Second, the long-term relationships among analyzed variables were confirmed by both models. In the case of point estimates, an increase in the sense of happiness at 1% level of significance will decrease the inequalities by 1.73%. For comparison, an increase in the sense of happiness, in the model for annual data, by 1% will decrease the inequalities by 1.08%. Therefore, it can be concluded that both values are comparable, and there is no big discrepancy between them. Most of the short-term relationships in the model for annual data were confirmed in the model for quarterly data. However, increasing the frequency of data resulted in the identification of several additional short-term relationships. For example, we can point out that the dimension of education not only reduces inequality but also increases the feeling of happiness. On the other hand, the dimension of the natural environment shows the existence of a relationship between material dimension and education dimension. The differences between model for annual data and model for quarterly data are visible in the values of the ECM parameters. For the model based on annual data, the average speed of adjustment ranged from 30.6% to 55.4%. For the model based on quarterly data, the speed of adjustment to long-term equilibrium ranges from 0.01% to 6.9% for the quarter. The difference is significant; however, it is an average value for 8 economies, which suggests that, in the future, a different method of analysis should be used.

Taking into account the discrepancies in the point estimates of short-term parameters between CEE economies, we can conclude that the relationships between the income inequalities and dimension of well-being should be analyzed from the point of view of an individual economy. This will permit skipping the averaging of the values for CEE economies and analyze the detailed relationships between the variables. It is also the further direction of our research.

## Conclusion and discussion

The ongoing discussion about how income inequality affects well-being is ambiguous, and results are mixed in the literature. The possible identified factors of this inconclusiveness are selection of variables used in the analysis, the length of the analyzed periods, methods used, and different definitions of well-being (subjective, objective, or overall measures). To shed more light on this issue, we combined the different dimensions of well-being and income inequalities and considered the panel ARDL model. The results of conducted analysis allowed us to identify long-run and short-run associations between income inequalities and the particular dimensions of well-being. What is more, not only relationships between unequal distribution of income and 5 dimensions of well-being but also interrelations between different dimensions of well-being per se were examined. The results of conducted research showed that there were many links and proved that a multidimensional analysis in this field is required. What is more, there were no such analyses conducted for the CEE countries in the period after the accession to the European Union (2004–2018).

Our results are in line with the majority of previous studies in the perspective of negative relationships between income inequalities and subjective well-being—happiness [19, 20, 28, 55]. However, contrary to the positive links in this field [36, 37], it also confirms the findings of the negative associations between unequal income distribution and health [7, 32, 39] and natural environment [40]. It, however, contradicts the findings of Grunewald who presented a positive relationship between income inequalities and CO_2_ emissions in high-income countries [41]. Because of these differing results found in various studies, we aimed to conduct an in-depth analysis of these relationships based on the ARDL panel model.

The results analysis based on dynamic panel models allowed us to identify long-run and short-run associations between income inequalities and dimensions of well-being. In the long run, all the dimensions of well-being significantly affected income inequalities. However, three dimensions of well-being negatively affected unequal distribution (health dimension, natural environment dimension, happiness dimension). Positive long-run relationships were observed between income inequalities and material dimension and education. When the short-run relationship between unequal distribution and well-being was analyzed, we identified a direct impact of education on income inequalities (Table 5, Scheme 1 in Fig 2). What is more, there were direct short-run relationships identified running from income inequalities to health and happiness. Also, the deviation from equilibrium in equations for health dimension and happiness are corrected in 36% and 55.4%, respectively, in the next year through a short-run fluctuation in income inequalities, material dimension, and health dimension.

An important issue for future research is the examination of the differences in relationships between income inequalities and well-being dimensions in the context of 8 CEE economies. The applied PMG estimator allowed us to study the long-term and short-term impacts, but the results are the average values in the group of 8 economies. On the other hand, we should consider whether these relationships among income inequalities and well-being dimensions varied. Individual analysis of each economy using time series methods may result in different patterns of transferring variability between well-being dimensions and income inequality.

## Supporting information

S1 File Data used in research. (XLSX)
